# Structural, Magnetic and Optical Properties of Gd and Co Co-Doped YFeO_3_ Nanopowders

**DOI:** 10.3390/ma12152423

**Published:** 2019-07-30

**Authors:** Meng Wang, Ting Wang

**Affiliations:** 1 School of Mechanical and Electrical Engineering, Shenzhen Polytechnic, Shenzhen 518055, China; 2 Shenzhen Key Laboratory of Advanced Materials, Department of Materials Science and Engineering, Shenzhen Graduate School, Harbin Institute of Technology, Shenzhen 518055, China

**Keywords:** multiferroics, low-temperature solid-state reaction, optical properties, magnetic properties, co-doping

## Abstract

YFeO_3_, YFe_0.95_Co_0.05_O_3_, Y_0.95_Gd_0.05_FeO_3_ and Y_1−x_Gd_x_Fe_0.95_Co_0.05_O_3_ (x = 0.0, 0.05, 0.10, 0.15 and 0.20) nanopowders were successfully fabricated via a low-temperature solid-state reaction technique. Results obtained using X-ray diffraction (XRD), scanning electron microscopy (SEM), and Raman spectra indicate that YFeO_3_ nanopowders with Gd^3+^ and Co^3+^ ions co-doping at Y and Fe-sites were fabricated at 800 °C in sizes below 50 nm, and a distorted structure was obtained. Magnetic hysteresis loop analyses illustrate that ferromagnetic behavior of YFeO_3_ nanopowders can be enhanced with the addition of Gd and Co. Whereas the maximum and remnant magnetization of the powders were found to be about 5.24 and 2.6 emu/g, respectively, the optical band gap was around 2.4 eV, proving that co-doped YFeO_3_ nanopowders have a strong capability to absorb visible light. Because both magnetic and optical properties of these materials are greatly improved with the addition of Gd and Co, one can expect the scope of their potential application in the magnetic and optical fields to increase.

## 1. Introduction

As one of the cutting edge multiferroic materials, AFeO_3_ (A = La, Y and Sc) materials have been the focus of industry research because of their couple orderings of ferroelectricity and anti-ferromagnetism. As a result, they have great potential application in data storage, information exchange, and 5G mobile phone systems [1]. YFeO_3_ is one of the most promising applications of rare earth AFeO_3_ materials [2]. YFeO_3_ has been reported to feature molecular ferroelectricity at low temperatures (10–40 K), good dielectric and magnetic properties [3,4,5,6], and is also becoming one of the most widely investigated multiferroic materials. Moreover, with a narrow optical gap (1.9–2.6 eV), its potential application as an optical material, especially decomposing organics should be considered for further study [7]. However, pristine YFeO_3_ is not easy to prepare as evident by problems such as the introduction of secondary phases and the hopping of charge between Fe^2+^ and Fe^3+^ [8,9,10], while its low magnetism characteristics [11] are possibly its main shortcoming. Studies show that an effective and efficient way [11,12,13] to overcome these problems, is by means of doping. Some scientists substituted Y by divalent and trivalent ions [14,15] and Fe by trivalent and high-valence ions [16]. While the use of Gd doping is rarely reported, one study showed that with Gd doping on the Y site, significant enhancement of magnetization was achieved. In addition, use of other-element doping on the Y site has the potential for improvement of the material’s optical properties [15]. For Fe-site ion doping, the main improvement focus was the reduction of leakage current and the enhancement of magnetic properties. It is known [17] that Y(Fe,Cr)O_3_ showed evidence of ferromagnetic property. Ma et al. [18] synthesized Mn doped YFeO_3_ by using a standard solid-state reaction method. Their findings showed that the leakage current was reduced so that ferroelectric properties are realized at room temperature.

Co doping may enhance the magnetic properties of RFeO_3_ material because it is magnetically active. While there is no report of co-doping of Co and Gd, the purpose of this paper is the study of co-doping of Co and Gd on YFeO_3_ with a particular emphasis on the microstructural, optical, and magnetic properties of the doped YFeO_3_ nanopowders.

## 2. Experimental Procedures

YFeO_3_ (abbreviated as YFO), YFe_0.95_Co_0.05_O_3_ (Co5), Y_0.95_Gd_0.05_FeO_3_ (Gd5), Y_0.95_Gd_0.05_Fe_0.95_Co_0.05_O_3_ (Gd5Co5), Y_0.9_Gd_0.1_Fe_0.95_Co_0.05_O_3_ (Gd10Co5), Y_0.85_Gd_0.15_Fe_0.95_Co_0.05_O_3_ (Gd15Co5), and Y_0.8_Gd_0.2_Fe_0.95_Co_0.05_O_3_ (Gd20Co5) nanoparticles were synthesized using a low-temperature solid-state reaction technique as described in Ref. [19]. The raw reagents include Fe(NO_3_)_3_∙9H_2_O, Y(NO_3_)_3_∙6H_2_O, Co(NO_3_)_2_·6H_2_O, Gd(NO_3_)_3_·6H_2_O, and citric acid. Initially, Fe(NO_3_)_3_∙9H_2_O, Y(NO_3_)_3_∙6H_2_O and citric acid were weighed stoichiometrically and ground in an agate mortar for half an hour, respectively. The respective powders were then mixed and ground again in an agate mortar for half an hour. A light brown viscous substance was formed during the grinding process, implying that the complex was formed. The viscous substance was heated at 120 °C for 2 h to remove free water and then a powdery composite was harvested, serving as the precursor powders. The powders were ground and subsequently calcined for 1 h in air at 800 °C to provide the nanoparticles. The crystal structures and morphology of the calcined particles were examined using XRD (D/max-RB, Rigaku, Tokyo, Japan) and SEM (S-4700, Hitachi, Tokyo, Japan). Raman spectra (InVia Reflex, Renishaw, London, UK spectrometer) were measured at room temperature with a range of 100 cm^−1^ to 1500 cm^−1^. Magnetic hysteresis loops were measured using PPMS (Physical Property Measurement System, DynaCool-9T, Quantum Design, Leatherhead, Surrey, UK). Optical absorbance properties of the experimental nanopowders were examined using a UV-Visible spectrophotometer (Shimadzu, UV-2600, Kyoto, Japan).

## 3. Results and Discussions

**Figure 1:**
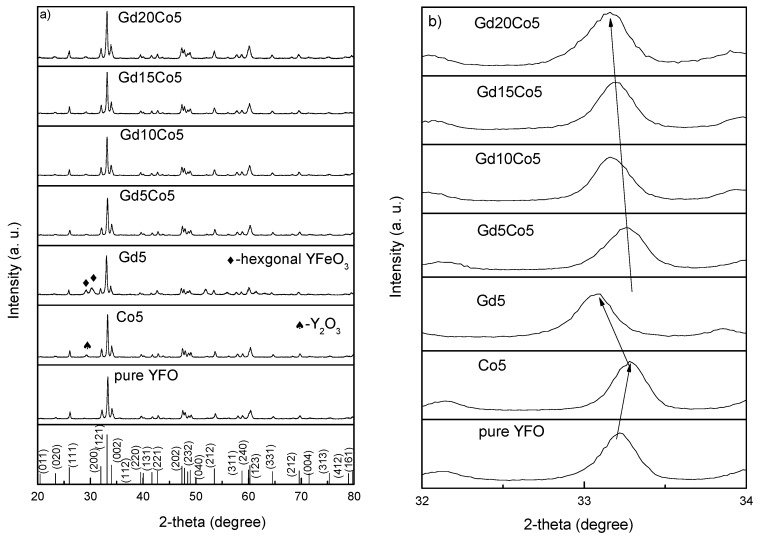
(**a**) XRD patterns of as-prepared nanoparticles calcined at 800 °C; (**b**) the magnified patterns of peaks at 2θ ~32°.

Figure 1a presents the XRD patterns of the tested (0 ≤ x ≤ 0.2) samples calcined at 800 °C. The pattern for the pristine YFeO_3_ nanopowders suggests the presence of the obvious orthorhombic YFeO_3_ pattern and that no minor impurity peaks were present. This outcome shows that the synthesis reaction for the orthorhombic structure was completed and the purity was high. The doped nanopowders indicate the same characteristic shape as that of the pristine YFeO_3_ patterns with a slight shift in the peak position and phase. For Gd5, some peaks for the hexagonal YFeO_3_ structure emerged. For Co5, peaks for Y_2_O_3_ appeared, showing that some minor impurity was introduced. This is a normal situation involving the case where instability of YFeO_3_ exists and which is due to the radius difference. However, this effect totally disappears for the Co-Gd co-doped sample. Figure 1b shows the YFeO_3_ peaks at 2θ ~33°, shifting toward a higher 2θ angle with substitution of Co into YFeO_3_. The figure also shows a shift towards a lower 2θ angle with further substitution of Gd into YFeO_3_, and while keeping almost the same position of Gd and Co-doped samples due to Gd^3+^ (Gd^3+^ 0.938 Å) possessed a larger ionic radii compared with Y^3+^ (Y^3+^ 0.9 Å), and Co^3+^ (Co^3+^ 0.61 Å) possessed a smaller one compared with Fe^3+^ (Fe^3+^ 0.645 Å) [15,20]. Thus, it is evident that Gd^3+^ and Co^3+^ replaced the Y^3+^ and Fe^3+^ ions in YFeO_3_, respectively. The XRD pattern shows the highest peak for the Co-doped particles and its 2θ angle location is nearly identical to that of pure YFO. The stress introduced by the change in radius was reduced so that impurities disappeared. 

Table 1 shows the lattice parameters with different Co and Gd concentrations. All these values increased with increase in Gd content and decreased with Co addition. This behavior demonstrates that the replacement of Y^3+^ with Gd^3+^ results in the increase of the lattice volume of YFeO_3_ (r_Gd_^3+^ > r_Y_^3+^), whereas the replacement of Fe^3+^ with Co^3+^ results in the decrease of YFeO_3_ volume (r_Co_^3+^ < r_Fe_^3+^). The continuous evolution of lattice parameters with increasing Gd and Co concentrations also shows the presence of a successful substitution of Y^3+^ and Fe^3+^ ions by Gd^3+^ and Co^3+^ ions.

**Figure 2:**
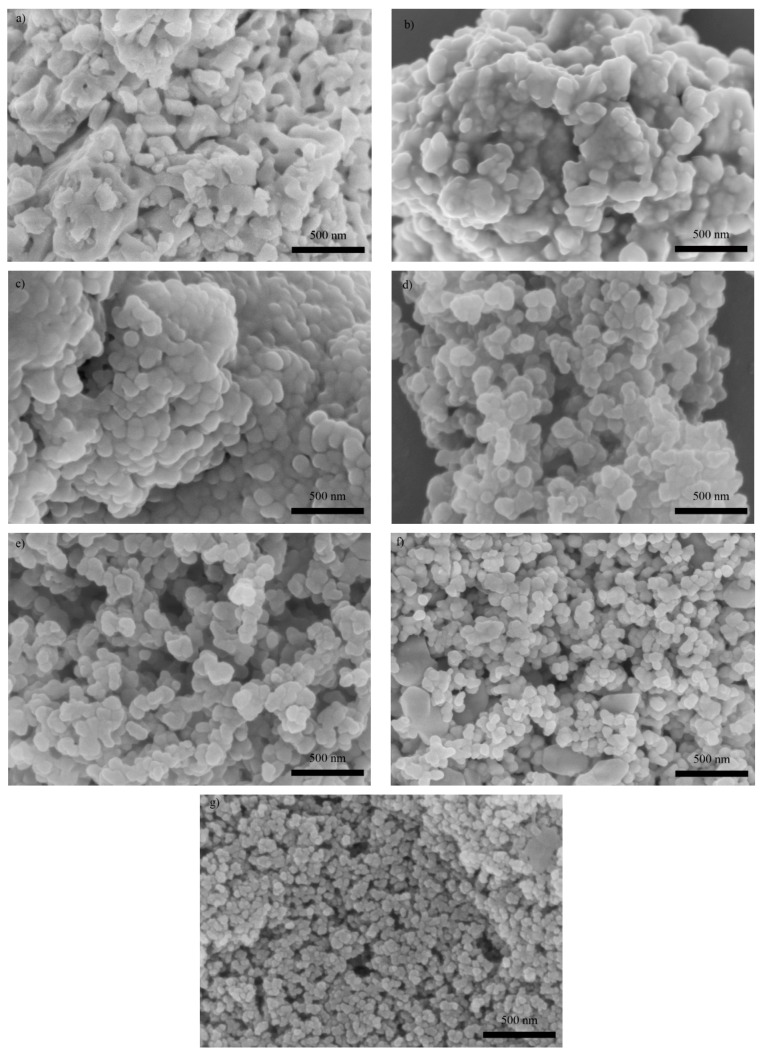
SEM micrographs of as-prepared nanoparticles: (**a**) YFO; (**b**) Co5; (**c**) Gd5; (**d**) Gd5Co5; (**e**) Gd10Co5; (**f**) Gd15Co5; (**g**) Gd20Co5.

SEM images of YFeO_3_ nanopowders are shown in Figure 2. These demonstrate that the particle sizes of the test nanopowders were homogeneous with some minor agglomeration. The particle sizes of the test samples of YFO, Co5, Gd5, Co5Gd5, Co5Gd10, Co5Gd15 and Co5Gd20 were approximately 150, 120, 95, 80, 75, 45 and 40 nm, respectively. From the results obtained, it is apparent that the Co and Gd substitution decreased the grain size, while particle size decreased significantly with Co and Gd co-doping. As is well known, whenever the diffusion rate is low, rare earth ions can inhibit grain growth of RFeO_3_ [11]. In addition, Gd^3+^ ions (radius of 0.983 Å) are larger than Y^3+^ ions (radius of 0.9 Å). The mismatch of ion sizes introduces defects in the lattice, leading to particle refinement. In addition, Co^3+^ can suppress hopping of Fe^2+^ and Fe^3+^, depriving the oxygen vacancy and subsequently preventing growth of the grains [21]. Some agglomeration of particles appears in Co and Gd co-doping samples, and may be attributed to the large surface area to volume ratio of the nanoparticles. The latter is to be expected whenever the low-temperature solid-state reaction method is used. Similar effects of grain refinement induced by rare earth and magnetic element co-doping has been reported in other references [14,17,21,22].

**Figure 3:**
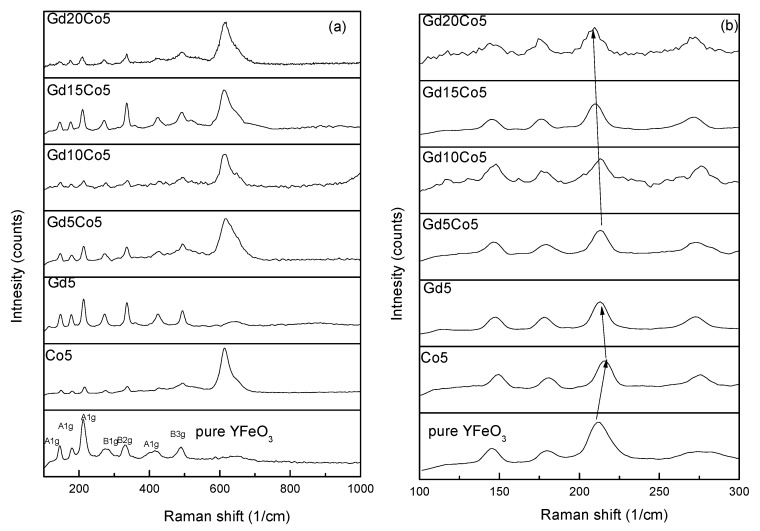
(**a**) Raman spectra of as-prepared nanoparticles. (**b**) The magnified patterns of 221 cm^−1^.

Figure 3 shows the Raman patterns obtained from the YFeO_3_ powders. As is well known, for the case involving the Pnma structure of YFeO_3_, only modes of A_1g_, B_1g_, B_2g_, and B_3g_ are active. Furthermore, one can say with a high degree of certainty that the first and second A_1g_ modes are associated with Y–O bonds, and the A_1g_ and B_g_ modes are related to Fe-O bonds at higher frequencies [23]. The 221 cm^−1^ A_1g_ mode is represented as an FeO_6_ octahedral structure [9,10,24]. The vibrating modes of Co5 were similar to those of pure YFO, while the intensity was reduced slightly and the peak at 610 cm^−1^ increased as expected owing to the minor change of Fe–O bonds resulting from the doping of the Co element. For the case involving substitution of Gd^3+^ for Y^3+^, the parameters of lattice (grain size) increased, leading to a shift in the Raman bands to a lower wave-number. Raman bands were found to broaden with increase of Gd^3+^ and Co^3+^ (in YFeO_3_) because of the disorder introduced by two different cations. The Gd–O and Y–O bonds, with their inherent different strengths, can influence their vibration frequencies. In general, one can state that these results are consistent with the XRD patterns. Thus, it can be concluded that Co and Gd co-doping has a substantial effect on the modification of the YFeO_3_ structure.

**Figure 4:**
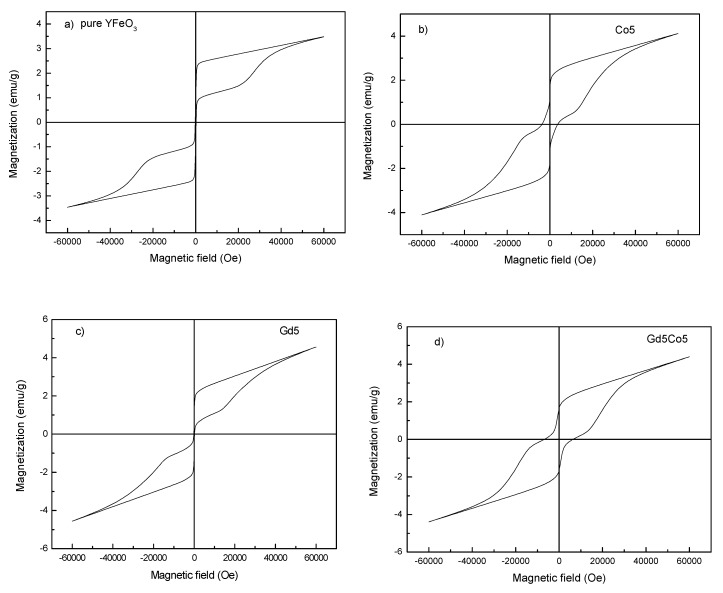

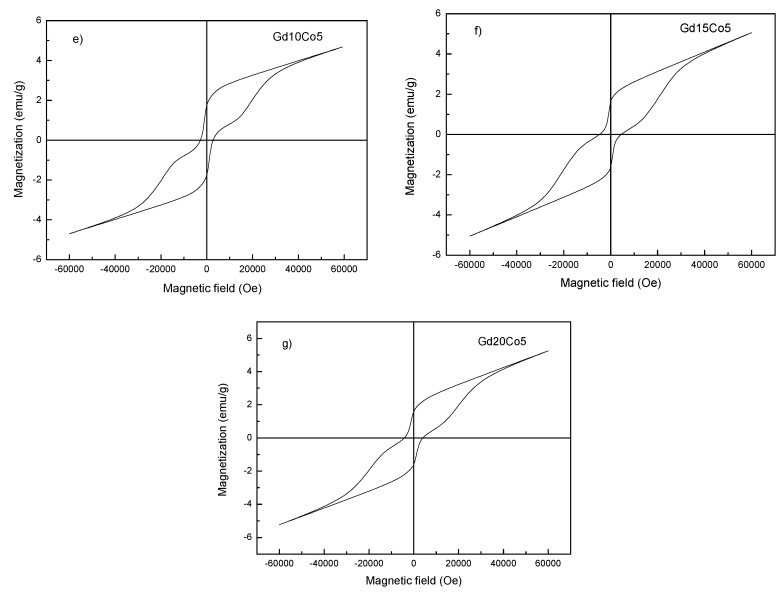
Magnetic hysteresis loops of as-prepared nanoparticles: (**a**) YFO; (**b**) Co5; (**c**) Gd5; (**d**) Gd5Co5; (**e**) Gd10Co5; (**f**) Gd15Co5; (**g**) Gd20Co5.

Figure 4 shows the magnetic properties of the nanopowders at room temperature. Values of maximum magnetization (*M_m_*), remnant magnetization (*M_r_*), and the coercive field (*H_c_*) of the test samples are listed in Table 2. As is commonly known, YFeO_3_ is antiferromagnetic and features weak magnetic properties. In the case of pristine YFeO_3_, the hysteresis loop is typical weak magnetization in antiferromagnetic type. The magnetization parameters for the pristine YFeO_3_ sample *M_m_*, *M_r,_* and *H_c_* had magnitudes of 3.50 emu/g, 0.89 emu/g, and 161 Oe. For the case involving the doped samples, a large open region was seen at the center of the hysteresis loops suggesting ferromagnetic behavior. Even when exposed to a 60 kOe outer magnetic field, the loops were not saturated. For Co5, *M_m_*, *M_r_* were slightly improved and *H_c_* was reduced. Their corresponding values were about 4.56 emu/g, 0.95 emu/g and 120 Oe, respectively. For the case of co-doping of Co and Gd, the net magnetization decreased slightly at first, and then began to increase, reaching a high value of 5.24 emu/g for the Co5Gd20 particles. These values are summarized in Table 2. The value of *M_r_* reached a maximum (*M_r_* = 1.66 emu/g) for the Co5Gd5 nanoparticles. The reasons for observed improvement of the magnetization may be summarized as follows: 

(1) The effect of nanoparticles. Uncompensated surface spins of Fe^3+^ ions are created when particle size is small. This situation leads to a strong magnetic enhancement [25,26].

(2) The distorted structure is affected by the doping effect. A FeO_6_ octahedron structure consists of one Fe^3+^ ion and six O^2−^ ions, while each Fe^3+^ magnetic moment is not precisely parallel to the neighboring ones, forming a small angle [25,26], which causes weak ferromagnetism in the antiferromagnetic YFeO_3_. Substituting an Fe^3+^ ion with a larger radius Co^3+^ ion will reduce the canted angle of the FeO_6_ octahedra and release the distortion, and combined with a refined powder size, a higher magnetization is achieved. [27].

(3) The Gd^3+^ ion with a large magnetic moment (μ_eff_ = 8.0 μ_B_) is magnetically active. When Y^3+^ is replaced by Gd^3+^, Y–O–Gd chains instead of Gd–O–Gd chains are formed, further improving the magnetization of the Co and Gd co-doped nanopowders [15].

In summary, the three reasons noted above serve to bring about the enhancement of magnetization. Others who conducted similar research made similar findings. Khalifa et al. [16] reported that *M_m_* and *M_r_* were about 0.8 emu/g and 0.1 emu/g for Ti-doped YFeO_3_ nanoparticles prepared using an improved sol-gel technique. Shi et al. [17] synthesized YFe_0.5_Cr_0.5_O_3_ nanoparticles by the sol-gel method, and showed that *M_m_* and *M_r_* were approximately 2.0 and 0.5 emu/g, respectively. As reported by Shi, the maximum *M_m_* and *M_r_* values for Y_0.95_Ho_0.05_Fe_0.5_Cr_0.5_O_3_ nanoparticles prepared using a low-temperature citric acid assisted sol-gel technology were about 4.5 and 1.2 emu/g, respectively [17]. Yuan et al. [15] synthesized Y_0.9_Gd_0.1_FeO_3_ using a solid-state reaction method, and obtained values of *M_m_*, *M_r_*, and *H_c_* of 2.5 emu/g, 1.0 emu/g and 30,000 Oe, respectively. Still, it is worth noting that the property values in the present study are comparable with, or better than the results reported by others.

**Figure 5:**
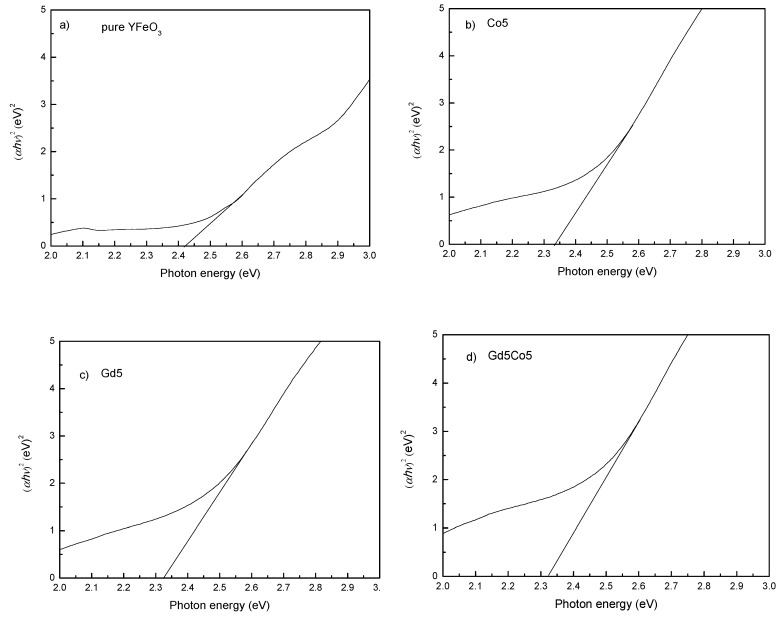

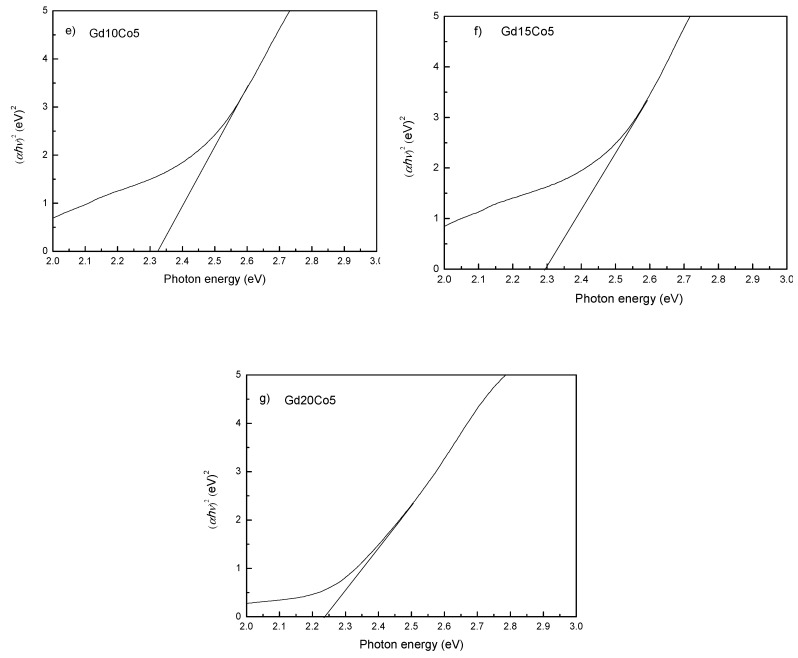
(*αhν*)^2^ as a function of photon energy for as-prepared nanoparticles: (**a**) YFO; (**b**) Co5; (**c**) Gd5; (**d**) Gd5Co5; (**e**) Gd10Co5; (**f**) Gd15Co5; (**g**) Gd20Co5.

YFeO_3_ possesses a narrow optical band gap (1.6–2.4 eV) and has been used in light-electric energy conversion applications. From the UV-visible absorption spectra (Figure 5), the optical energy band gap (*E_g_*) of the seven test samples can be calculated using a Tauc function expressed in Equation (1) [28]:

(1) $${\left(\alpha hv\right)}^{n}=A\left(hv-E_{g}\right)$$

The *E_g_* values shown in Figure 5 illustrate strong visible light absorption, indicating their promising decomposition application (See Table 2). YFO has the maximum *E_g_* value (2.42 eV). It is decreased to 2.15 eV for Gd20Co5 nanoparticles. Clearly, the energy band gap becomes smaller with refined particle size of the YFeO_3_ powders. Thus, the reduction of the energy gap with co-doping is ascribed to the smaller particle size and lattice distortion reduction generated by the addition of Co and Gd. Further, according to Reference [23], reduced particle size leads to a narrow energy gap. Nonuniform microstrains caused by lattice distortions can impact energy levels, affecting the energy band gap [28,29]. YFeO_3_ possesses the band gap of 2p O, and 4d Y atoms. All these states are partially filled [28]. The Gd atom is with the 4f state which is also not fully filled orbits. Thus f-d hybridization of the 4d Y and 3d Fe atoms causes light absorption. In addition, the same d states of Fe and Co overlap with the 2p states O atom, causing a narrow energy gap [15,30]. Moreover, Gd^3+^ and Co^3+^ ions partially substituting Y^3+^ and Fe^3+^ ions refine the as-synthesized nanoparticles size and increase chemical pressure, thereby resulting in a smaller value of *E_g_* [30]. Zhang et al. [28] obtained *E_g_* values of 1.94, 2.43 and 2.30 eV, respectively, for the hexagonal, orthorhombic, and YFeO_3_ containing a mixture of the two phases. Shen et al. [31] reported that the *E_g_* value obtained from a first-principles calculation for YFeO_3_ ceramics was 2.58 eV. Wu et al. [23] prepared YFeO_3_ in which, hexagonal and orthorhombic phases co-existed; this material had a band gap of 2.41 eV. For YFeO_3_ ceramics prepared by the conventional solid-state method, the energy band gap was found to be 2.58 eV [7]. Liu et al. [32] measured the optical properties of hexagonal-YFeO_3_/α-Fe_2_O_3_ heterojunction composite nanowire and obtained *E_g_* values of approximately 2.15 eV. Once again, it is worth noting that our *E_g_* values compare favorably with these results. Therefore, one can reasonably conclude that the YFeO_3_ nanopowders used in this study can be used in the decomposition of organic compounds.

## 4. Conclusions

Co and Gd co-doped YFeO_3_ nanopowders were fabricated using a low-temperature solid-state reaction method. Data obtained using XRD and Raman analyses show that, with Gd and Co substitution, YFeO_3_ nanoparticles exhibit a distortion refined structure. The particle size for pristine YFeO_3_ nanoparticles is about 100-200 nm, and those for Co5, Gd5, Co5Gd5, Co5Gd10, Co5Gd15 and Co5Gd20 are approximately 120, 95, 80, 75, 45 and 40 nm, respectively. The maximum magnetization and remnant magnetization for the co-doped YFeO_3_ powders are about 5.49 and 2.20 emu/g, respectively, when exposed to a magnetic field of 60 kOe. The energy band gap of YFeO_3_ nanopowders was reduced from 2.41 to 2.23 using the co-doping method, thereby indicating their potential in decomposition applications. Because the co-doping method used in the study was found to be easy to control for fabricating the YFeO_3_ nanopowder samples, it is proposed that the method be adopted for use in the applied magnetic and optical fields.

## Figures and Tables

**Table 1:** Lattice parameters and volume of the samples.

Sample	a (Å)	b (Å)	c (Å)	Volume (Å ^3^)
Pure YFO	5.58079	5.27096	7.58866	223.23
Co5	5.57756	5.26958	7.58258	222.86
Gd5	5.58195	5.27209	7.5924	223.43
Gd5Co5	5.58018	5.27106	7.58943	223.23
Gd10Co5	5.58255	5.27279	7.59356	223.52
Gd15Co5	5.58303	5.27411	7.59364	223.60
Gd20Co5	5.58523	5.27791	7.59615	223.92

**Table 2:** List of the magnetic and optical parameters for YFeO_3_ powders.

Sample	*M_m_* (emu/g)	*M_r_* (emu/g)	*H_c_* (kOe)	Photon Energy (eV)
Pure YFO	3.49	0.88	160	2.42
Co5	4.10	1.47	3700	2.34
Gd5	4.56	0.84	170	2.33
Gd5Co5	4.40	1.66	6800	2.32
Gd10Co5	4.68	1.76	2800	2.32
Gd15Co5	5.05	1.62	4800	2.29
Gd20Co5	5.24	1.60	4200	2.24
